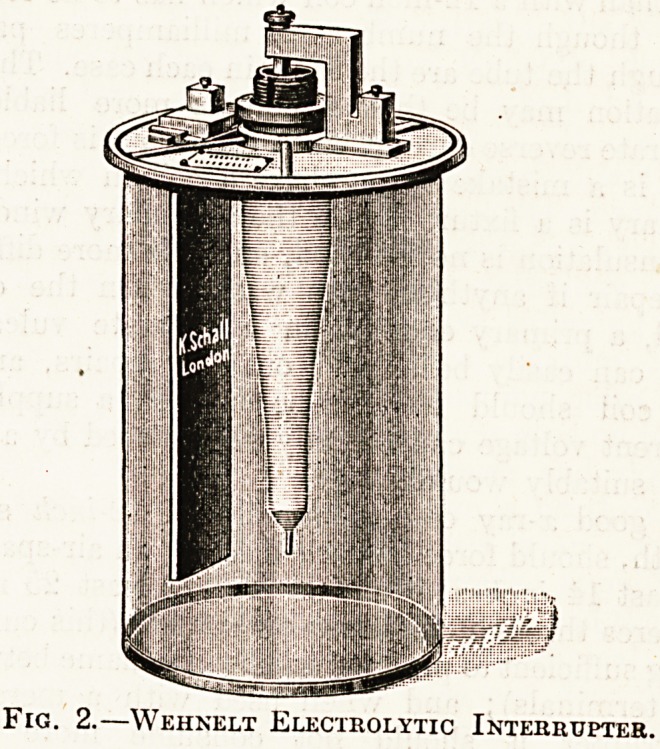# The X-Rays

**Published:** 1912-05-04

**Authors:** Alfred C. Norman

**Affiliations:** House Surgeon at Sunderland and Durham County Eye Infirmary, Sunderland.


### ELECTRICITY IN MODERN MEDICINE.*

### XI.—The X-Rays.

By ALFRED C. NORMAN, M.D. Edin., House Surgeon at Sunderland and Durham County Eye Infirmary, Sunderland.

The primary winding of an induction coil must be specially designed to suit the voltage of the current on which it is to be worked. Many of the old coils intended for use with 12-volt accumulators can now be used on the 100-volt lighting mains by inserting suitable resistance in the primary circuit, but their efficiency must necessarily be very low.

A so-called 12-*inch* coil is the smallest size advisable for general *x*-ray work, but this size will do all that most people ever require of it. Where expense need not be considered, however, a 16- or even a 20-inch coil should be obtained, and for instantaneous work these larger sizes are very advantageous. The writer's experience has been almost entirely confined to 12-inch coils, and he has always been able to obtain as much current from them as his tubes will stand. There is no doubt, however, that a tube has a longer life if used with a 16- or 20-inch coil working well below its maximum output than with a 12-inch coil which has to be forced, even though the number of milliamperes passed through the tube are the same in each case. The explanation may be that a coil is more liable to generate reverse current when its output is forced.

It is a mistake to purchase a coil in which the primary is a fixture inside the secondary winding; the insulation is not so good, and it is more difficult to repair if anything goes wrong. On the other hand, a primary encased in a separate vulcanite tube can easily be slipped out for repairs, and if the coil should have to be used on a supply of different voltage can actually be replaced by a new one, suitably wound.

A good *x*-ray coil, nominally of 12-*inch* spark length, should force a spark through an air-space of at least 14 *inches*; it should send at least 25 milliamperes through an air-gap of 8 inches (this current being sufficient to produce an absolute flame between the terminals); and when used with a mercury-interrupter it should not consume more than 5 amperes in the primary circuit in giving these results. With such a coil a fully exposed radiogram of a shoulder can be obtained in about 20 seconds and of an average pelvis in less than one minute. It costs about £25, complete with condenser.

### The Interrupter.

The most primitive type of interrupter is the Neef's (or Wagner's) hammer, familiar to all of us in the days of our physics and physiology classes. This hammer is fixed to the base of the coil, and oscillates between two contacts as a result of counter forces supplied by a steel spring and the magnetic attraction of an iron core respectively. These interrupters are still used on medical coils for faradic treatment, but for *x*-ray work, where greater amperage and much higher voltages have to be employed in the primary circuit, they have long been superseded by a more complicated piece of mechanism quite separate from the induction coil. The trouble with the hammer interrupters was that they could not be made to work under paraffin or some other di-electric which would damp the spark, so that the platinum contacts were soon burnt away by the large currents; also they were noisy, and were not rapid enough for modern requirements.

Modern interrupters may be divided into two classes—motor and electrolytic; the former is best suited to general requirements, and will be considered at greater length.

Whatever the type of interrupter its function is to rapidly make and break the primary circuit at least fifty times per second with as little sparking and as little noise as possible. It does not matter at what part of the circuit the interruption takes place, the result is the same. One of the conducting wires (whether positive or negative is immaterial) is cut, and the cut ends are connected with opposite sides of the interrupter.

The first motor interrupter to be introduced was the mercury dipper. In this type one end of the cut wire was connected with some mercury in the bottom of a glass vessel and the other with a metal rod. By means of a motor with an eccentric bearing the rod was rapidly dipped into and out of the mercury, thus making and breaking the primary circuit. To suppress the sparking at break a pint or so of methylated spirit was placed in the glass vessel above the level of the mercury. These interrupters are not sufficiently rapid nor will they pass the large currents required for modern radiography.

The next type to be introduced was the Mackenzie Davidson interrupter, in which a triangular copper sector fixed to an oblique axle was rapidly rotated by a motor over a vessel of mercury, in such a way that the point of the sector made contact with the mercury once per revolution. One of the cut ends of the conducting wire was connected with the metal vessel containing mercury, the other with a spring contact on the axle; when the sector touched the mercury the primary circuit was made, and during the rest of the revolution it was broken. Methylated spirit was used above the mercury to damp out the sparking at break. The new pattern Mackenzie Davidson interrupter, in which the metal sector is replaced by a disc or wheel of insulating fibre containing two metal segments, is still used with satisfaction by many workers. The lower edge of the fibre wheel dips into the mercury, and as it rotates it brings the two metallic segments alternately into contact with the mercury, and thus the circuit is made and broken twice per revolution of the axle. Instead of a methylated spirit di-electric the vessel is made air-tight and is kept full of coal-

* Previous articles appeared on Nov. 11 and 25, Dec. 9 and 30, Jan. 13 and 27, Feb. 17, March 9 and 30, April 20.

gas, which damps the spark just as satisfactorily and is much cleaner in use.

The next to be introduced was the mercury jet interrupter, in which the metal segments remain stationary, contact being made and broken by a thin jet of mercury pumped up by centrifugal force and rapidly revolved against the segments. Coal-gas or methylated spirit can be used to damp the spark, and as many as 5,000 interruptions per minute can be obtained owing to the fact that four contacts per revolution are made.

The latest, and in the writer's opinion the most efficient, interrupter is the centrifugal (fig. 1), and this is the interrupter which works best with the installation we are discussing. It is constructed as follows: A pear-shaped metal bowl is directly coupled to the armature of an electric motor. Some mercury is placed in the bottom of the bowl, and at

Fig. 1.—Centrifugal Mercury Interrupter.

the widest part of the inside of the bowl there is a deep groove extending horizontally round its whole circumference. When the bowl is revolved on a vertical axis mercury travels up its sides by centrifugal force and forms a complete ring round its widest part—*i.e.* the groove. A small fibre wheel with two metal segments is held eccentrically in the bowl, by a steel axle, in such a way that the periphery of the wheel presses into the mercury in the groove and rotates with it. But since the wheel is smaller than the bowl it revolves much more rapidly, and since the metal segments touch the mercury twice per revolution of the fibre wheel this type of interrupter will give from 8,000 to 10,000 breaks per second without overrunning the motor. Paraffin oil is the di-electric used to suppress the spark, and it does so very completely.

The advantages of the centrifugal interrupter are as follows: The contact is very perfect, hence it will transmit exceptionally large currents and will break them efficiently. The mercury is not churned into an emulsion. The oxide which usually fouls a mercury break is separated from the mercury by centrifugal action, hence the apparatus can be used for more than a year without requiring to be cleaned. The duration of contact—*i.e.* the length of time during which the primary current is made can be controlled by means of a screw which presses the wheel deeper into the mercury or withdraws it at will. And, finally, this type is by far the most rapid of all the mechanical interrupters.

We have seen that all modern mechanical interrupters are driven by a motor. The motor circuit is, of course, quite independent of the interrupter circuit proper and should be controlled by a small rheostat on the switchboard, so that the speed of the motor, and consequently the number of interruptions per second, can be accurately regulated.

The Wehnelt electrolytic interrupter (fig. 2) depends upon an entirely different principle. A thin platinum wire and a thick lead electrode are immersed in a solution of sulphuric acid. When current is passed through this solution from the platinum wire to the lead plate—*i.e. so that the wire is*

Fig. 2.—Wehnelt Electrolytic Interrupter.

*the positive electrode*—the current density at the point of the wire is so great that steam is formed and the water is split up into gases. These gases are rapidly formed and rapidly disappear, and in doing so they make and break the primary current very efficiently. An electrolytic break is the most rapid of all breaks, and it will transmit more current than any other type, but it has too many disadvantages ever to become popular except in the hands of experts. It is noisy; it gives off acid fumes which corrode other instruments in the *x*-ray room (hence it should be kept in another room and be connected with coil and switchboard by long cables). It requires careful tuning up before it will work. It is liable to stop working when the electrolyte becomes heated. And, finally, it consumes far more current for a given output than a mercury interrupter.

(*To be continued.*)

## Figures and Tables

**Fig. 1. f1:**
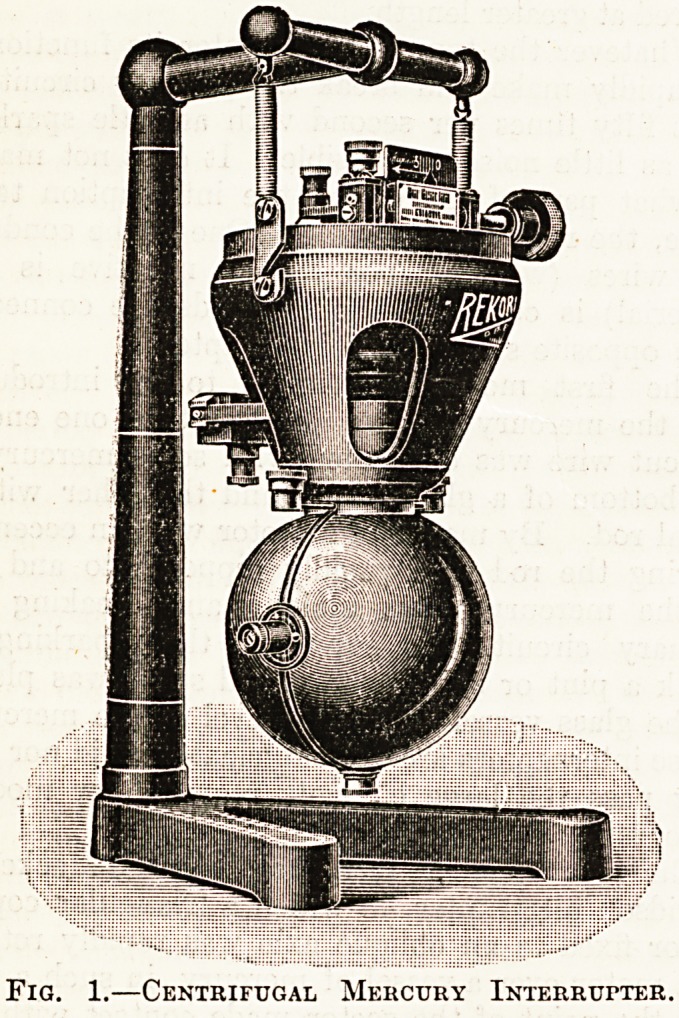


**Fig. 2. f2:**